# The effect of ozone exposure on the release of eicosanoids in guinea-pig BAL fluid in relation to cellular damage and inflammation

**DOI:** 10.1080/09629359791497

**Published:** 1997-12

**Authors:** H. J. M. Van Hoof, F. J. Zijlstra, H-P. Voss, I. M. Garrelds, J. A. M. A. Dormans, L. Van Bree, A. Bast

**Affiliations:** 1 Leiden/Amsterdam Center for Drug Research Department of Pharmacochemistry Vrije Universiteit De Boelelaan 1083 Amsterdam 1081 HV The Netherlands; 2 Department of Toxic Effects Laboratory of Health Effects Research National Institute of Public Health and the Environment P.O. Box 1 Bilthoven 3720 BA The Netherlands; 3 Laboratory of Pathology and Immunobiology National Institute of Public Health and the Environment P.O. Box 1 Bilthoven 3720 BA The Netherlands; 4 Department of Pharmacology Faculty of Medicine Erasmus University P.O. Box 1738 Rotterdam 3000 DR The Netherlands

## Abstract

The observed effects after ozone exposure strongly depend on ozone concentration and exposure time. We hypothesized that depending on the O_3_ exposure protocol, mainly either an oxidant damage or an inflammation will determine the O_3_ toxicity. We compared two different ozone exposure protocols: an acute exposure (3 ppm 2 h) for studying the oxidant damage and an exposure (1 ppm 12 h) where an inflammatory component is also probably involved. We measured LDH activity and protein and albumin exudation as markers for cellular damage. After the acute exposure an increase in LDH activity was measured and after exposure to 1 ppm ozone for 12 h the exudation of protein and albumin was also enhanced. The histological examinations showed a neutrophilic inflammatory response only after exposure to 1 ppm ozone for 12 h. The acute exposure protocol resulted in an increased release of PGE_2_, PGD_2_, PGF_2α_ and 6-ketoPGF_1α_ whereas exposure to 1 ppm ozone for 12 h led to an additional release of LTB_4_. No effects were measured on the release of TxB_2_ and LTC_4_/D_4_/E_4_. These changed amounts of eicosanoids will probably contribute to the ozone-induced lung function changes.


Mediators of Inflammation, 6, 355–361 (1997)

### The effect of ozone exposure on the release of eicosanoids in guinea-pig BAL fluid in relation to cellular damage and inflammation

H. J. M. van Hoof[1,2,CA], F. J. Zijlstra[4], H-P. Voss[1], I. M. Garrelds[4], J. A. M. A. Dormans[3], L. van Bree[2] and A. Bast[1]

[1]Leiden/Amsterdam Center for Drug Research, Department of Pharmacochemistry, Vrije Universiteit, De Boelelaan 1083, 1081 HV Amsterdam; [2]Department of Toxic Effects, Laboratory of Health Effects Research and [3]Laboratory of Pathology and Immunobiology, National Institute of Public Health and the Environment, P.O. Box 1, 3720 BA Bilthoven; [4]Department of Pharmacology, Faculty of Medicine, Erasmus University, P.O. Box 1738, 3000 DR Rotterdam, The Netherlands

[CA]Corresponding Author
Tel: (+31) 20 444 7587/7585
Fax: (+31) 20 444 7610
Email: vanhoof@chem.vu.nl

**The observed effects after ozone exposure strongly depend on ozone concentration and exposure time. We hypothesized that depending on the $O_3$ exposure protocol, mainly either an oxidant damage or an inflammation will determine the $O_3$ toxicity. We compared two different ozone exposure protocols: an acute exposure (3 ppm 2 h) for studying the oxidant damage and an exposure (1 ppm 12 h) where an inflammatory component is also probably involved. We measured LDH activity and protein and albumin exudation as markers for cellular damage. After the acute exposure an increase in LDH activity was measured and after exposure to 1 ppm ozone for 12 h the exudation of protein and albumin was also enhanced. The histological examinations showed a neutrophilic inflammatory response only after exposure to 1 ppm ozone for 12 h. The acute exposure protocol resulted in an increased release of $PGE_2$, $PGD_2$, $PGF_{2\alpha}$ and 6-keto$PGF_{1\alpha}$ whereas exposure to 1 ppm ozone for 12 h led to an additional release of $LTB_4$. No effects were measured on the release of $TxB_2$ and $LTC_4/D_4/E_4$. These changed amounts of eicosanoids will probably contribute to the ozone-induced lung function changes.**

**Key words:** Ozone, Guinea pigs, Inflammation, Neutrophils, Eicosanoids

### Introduction

Ozone is an important constituent of photochemical smog and because of its high oxidation potential is able to produce alterations in various functional, biochemical and morphological properties in the airways of humans and experimental animals.[1] Cellular membranes are sensitive to oxidative stress because they contain important targets like polyunsaturated fatty acids, sulfhydryl groups and amino acids of proteins which can easily be oxidized. Damage to cellular membranes may result in the release of lactate dehydrogenase (LDH) and an accumulation of proteins and inflammatory cells in broncho-alveolar lavage (BAL) fluid which are generally seen as biomarkers for lung injury. Also after exposure to ozone these phenomena are observed in animal as well as in human studies.[2–7]

Prostaglandins and leukotrienes have been shown to be importantly involved in the inflammatory response in the airways[8–12] and also some studies have been performed to investigate the role of these lipid mediators in the ozone-induced lung function changes.[4,13–16] However, due to relatively large interspecies differences[17–20] concerning receptor distribution and the release of eicosanoids the obtained results are rather contradictory. Also the exposure protocols used differ widely among the studies performed to investigate the effects of ozone exposure. The main purpose of the studies carried out in our laboratories is to unravel the complexity underlying the ozone-induced changes in guinea-pig lung function. The current study deals with the release of eicosanoids in guinea-pig BAL fluid in relation to the inflammatory response. The results presented here come from two different ozone exposure protocols; an acute exposure to 3 ppm ozone for 2 h where probably only an oxidative effect is responsible for the observed damage and an exposure to a lower ozone concentration (1 ppm) over a longer period of time (12 h) where the effects are expected to

© 1997 Rapid Science Publishers 

be mainly determined by an inflammatory response. Our objective is to study and compare the release of eicosanoids in both the ozone exposure protocols i.e. oxidant and inflammation mediated toxicity.

### Materials and Methods

### Animals

Male Dunkin-Hartley guinea pigs, weighing 300–350 g, obtained from Harlan CPB (Zeist, The Netherlands) were kept in a light- and temperature-controlled room (21 ± 1°C, humidity 50 ± 5%). The animals were fed a standard diet (Hope Farms, Woerden, The Netherlands) and were allowed to tap water *ad libitum*. The animals were adapted to the laboratory housing conditions for at least 1 week before starting the exposure.

### Animal exposure

Guinea pigs were placed separately in rectangular stainless steel inhalation chambers[21] with a volume of 0.21 $m^3$. Two different exposure conditions were used: exposure to 3 ppm (6 mg/$m^3$) ozone for 2 h and exposure to 1 ppm (2 mg/$m^3$) ozone for 12 h (guinea pigs were killed immediately after exposure). The ozone was generated by passing $O_2$ (pressure 1 atm.) through a pen-ray UV-light generator type 3 SC-9 with a SCT-4 power-supply (Ultra-Violet Products, San Gabriel, CA, USA) in which oxygen is partially converted to ozone. The ozone was diluted with filtered clean air as it was drawn into the exposure chamber with an air flow of 6.0 $m^3$/h. The exposure chambers were conditioned at a temperature of 21 ± 1°C and a relative humidity of 50 ± 5% The ozone concentration within each chamber was constantly monitored using a UV-Photometric analyzer model 9810 (Monitor Labs, San Diego, CA, USA). Calibration was performed before the exposure period using a UV-photometric calibrator (Thermo Environmental Instruments, Franklin, MA, USA). To maintain the ozone concentration at the desired value, a manual control was performed (Mass Flow Controller Type AFC-260, ASM, Bilthoven, The Netherlands).

Control animals were exposed under identical conditions to clean filtered air.

### Broncho-alveolar lavage (BAL)

After exposure to ozone lungs were removed, weighed and perfused using saline to remove excessive blood. Lungs were lavaged using 40 ml prewarmed (37°C) 0.9% saline per kg body weight. Three repetitive lavages with the same aliquot were performed by steady instillation and withdrawal through a tracheal cannula. The obtained BAL fluid was centrifuged for 10 min at 400 × ***g*** at 4°C and the cell-free lavage fluid supernatant was used for further analysis.

### Analysis of LDH, protein and albumin

Cell-free BAL fluid was analysed for total protein, albumin[22] and lactate dehydrogenase (LDH). Total protein was measured using the bicinchoninic acid (BCA) protein assay reagent according to the manufacturer's instructions (Pierce, Rockford, IL, USA). LDH activity was assayed at 37°C in 50 mM Tris-buffer, pH 7.4, with 5 mM EDTA, 0.15 mM NADH and 1.22 mM pyruvate by measuring the decrease in absorbance at 340 nm.

### Analysis of cyclooxygenase and lipoxygenase products from guinea-pig BAL fluid

The release of cyclooxygenase products was quantified using radioimmunoassays (RIA) for $PGE_2$, $PGF_{2\alpha}$, $PGD_2$, 6-keto$PGF_{1\alpha}$ and $TxB_2$ (antibodies from PerSeptive Diagnostics (USA), tritiated compounds from Amersham (UK), and standards from Sigma Chemicals (Belgium)) according to the manufacturer's instructions. Lipoxygenase products ($LTB_4$ and $LTC_4$/$D_4$/$E_4$) were measured using an enzyme immunoassay (EIA) system (Amersham, UK), according to the manufacturer's instructions. Samples were assayed in duplicate and standard curves were run with each assay of unknown samples. The production of cyclooxygenase and lipoxygenase products was expressed as picograms per millilitre (pg/ml) BAL fluid.

### Histological examination

After exposure to ozone the lungs were removed and inflated with a 2% solution of glutaraldehyde in 0.1 M phosphate buffer for fixation. After embedding in paraffin, 5 μm lung sections were stained with haematoxilin and eosin and examined by light microscopy.

### Data analysis

Each experiment was performed in duplicate and results were statistically evaluated using Student's *t*-test. $P < 0{\cdot}05$ was considered significant.

### Results

### LDH, protein and albumin measurements

Both exposure protocols (i.e. 3 ppm ozone for 2 h and 1 ppm ozone for 12 h) increased the amount of lactate dehydrogenase as measured in the guinea-pig BAL fluid (Table 1). The amount of total protein, or more specifically albumin, measured in the BAL fluid was not changed after exposure to 3 ppm ozone for 2 h but a significant increase was measured after exposure to 1 ppm ozone for 12 h.

### Leukotriene and prostaglandin measurements

After exposure to 3 ppm ozone for 2 h the release of $PGF_{2\alpha}$, $PGE_2$, $PGD_2$ and 6-keto$PGF_{1\alpha}$ was increased significantly compared with their respective controls ($PGF_{2\alpha}$: 1156 ± 122 pg/ml vs. 194 ± 42 pg/ml, $PGE_2$: 224 ± 20 pg/ml vs. 20 ± 5 pg/ml, $PGD_2$: 133 ± 26 pg/ml vs. 0 ± 0 pg/ml (not detectable) and 6-keto$PGF_{1\alpha}$: 832 ± 103 pg/ml vs. 428 ± 50 pg/ml) (Fig. 1). After exposure to 1 ppm ozone for 12 h comparable effects were measured ($PGF_{2\alpha}$: 831 ± 87 pg/ml vs. 181 ± 21 pg/ml; $PGE_2$: 161 ± 18 pg/ml vs. 39 ± 10 pg/ml; $PGD_2$: 117 ± 10 pg/ml vs. 0 ± 0 pg/ml (not detectable) and 6-keto$PGF_{1\alpha}$: 1210 ± 170 pg/ml vs. 604 ± 73 pg/ml) but after this exposure protocol also the amount of $LTB_4$ in the BAL fluid was increased (31 ± 2 pg/ml vs. 10 ± 3 pg/ml). No effects were measured on the release of $TxA_2$ (measured as $TxB_2$: 4231 ± 730 pg/ml vs. 4336 ± 1262 pg/ml (3 ppm 2 h) and 4494 ± 310 pg/ml vs. 3402 ± 811 pg/ml (1 ppm 12 h) and $LTC_4/D_4/E_4$ (46 ± 7 pg/ml vs. 44 ± 5 pg/ml (3 ppm 2 h) and 45 ± 12 pg/ml vs. 35 ± 6 pg/ml (1 ppm 12 h)).

**Table 1.** The effect of ozone exposure (3 ppm ozone for 2 h and 1 ppm ozone for 12 h) on cellular damage markers as measured in guinea-pig BAL fluid. Values represent mean ± SEM ($n = 5$) and *indicates $P < 0{\cdot}05$

| | LDH (U/l) | Total protein (mg/l) | Albumin (mg/l) |
|---|---|---|---|
| 3 ppm, 2 h | | | |
| Control | 72.4 ± 7.5 | 632 ± 118 | 347 ± 69 |
| Ozone exposed | 262.2 ± 17.9* | 831 ± 33 | 379 ± 13 |
| 1 ppm, 12 h | | | |
| Control | 106.4 ± 9.0 | 495 ± 88 | 221 ± 40 |
| Ozone exposed | 334.0 ± 32.7* | 1677 ± 204* | 566 ± 70* |

### Histological examination

In Fig. 2 the histological changes in guinea-pig lung tissue after ozone exposure are shown. In the control situation (Fig. 2A) a bronchiole, covered with cuboidal epithelium, is shown in cross-section together with expanded alveoli separated from each other by thin septa. Exposure to 3 ppm ozone for 2 h (Fig. 2B) results in a desquamation of the bronchiolar epithelium and thus a naked basement membrane. The bronchiolar lumen is filled with these desquamated epithelial cells. The alveolar lumina are empty and the alveolar septa are thin. A bronchiolitis consisting of polymorphonuclear cells (PMNs) and mononuclear cells is accompanied with a centriacinar inflammation when the animals are exposed to 1 ppm ozone for 12 h. The bronchiolar epithelial layer is intact (Fig. 2C).

### Discussion

It is shown in this and other studies that exposure to ozone is attended by an inflammatory response.[3–7] In this study we compared two different ozone exposure protocols (acute exposure to 3 ppm ozone for 2 h and an exposure to a lower concentration for a longer period of time (1 ppm ozone for 12 h) with respect to the inflammatory response coupled to the release of eicosanoids in the guinea-pig BAL fluid.

It is shown that the increase in the lactate dehydrogenase (LDH) activity can be seen as an early marker for ozone toxicity. LDH activity is the only biochemical marker we tested that already is affected after the acute exposure to ozone (3 ppm, 2 h). Measuring LDH activity represents an early indication for cellular damage; a slight change in membrane fluidity already causes a leakage of LDH from the alveolar cells in the lower airways. This rapid change in LDH release was also observed in an *in vitro* system using different types of cultured respiratory epithelial cells.[23] Exposure to relatively low ozone concentrations (0.5 ppm ozone for 3 h) already caused membrane injury of the epithelial cell types leading to increased lactate dehydrogenase release. Also in human studies early changes in LDH-release were measured in BAL-fluid[24,25] after ozone exposure.

The other two markers for cellular damage, albumin and protein, indicate an increased permeability leading to exudation of the constituents from serum into the airways. This exudation was measurable only after exposure to 1 ppm ozone for 12 h. The increase in serum

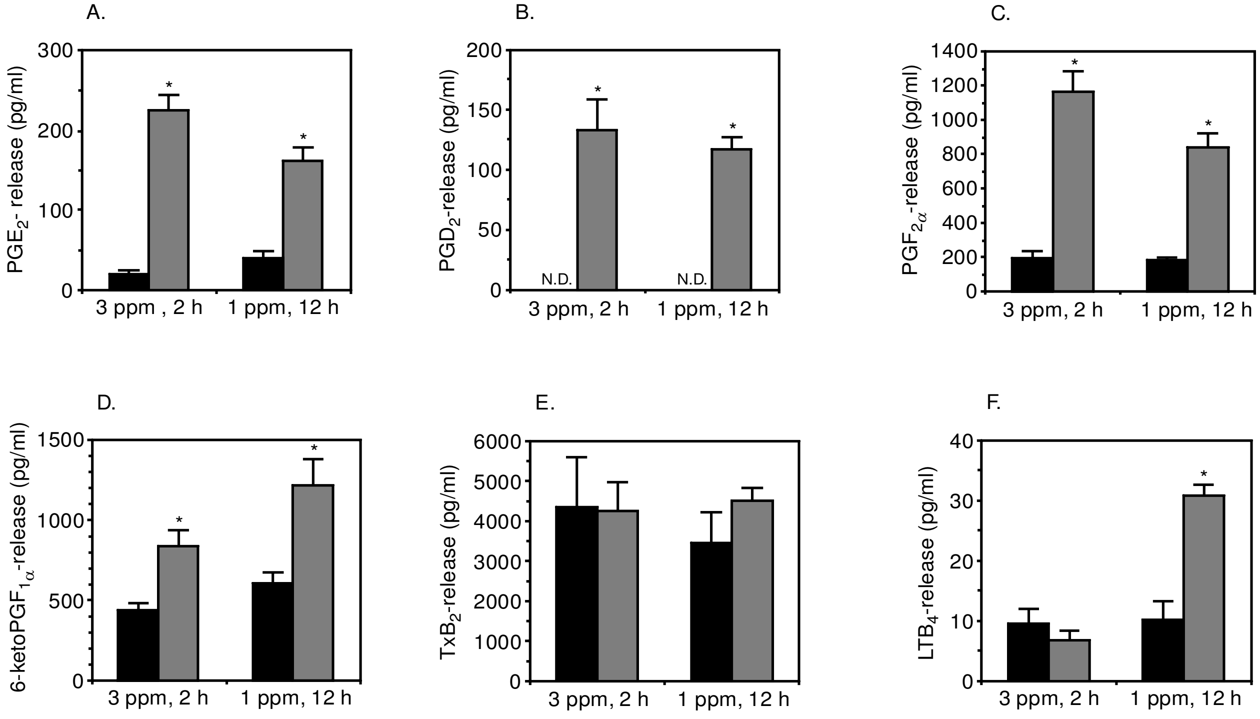

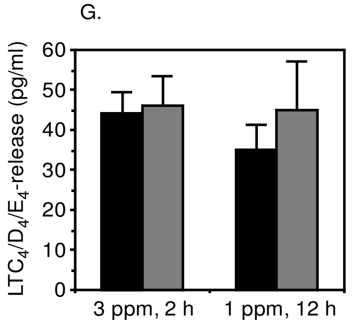

FIG. 1. The effect of ozone exposure on the release of the different eicosanoids from guinea-pig BAL fluid ((A) $PGE_2$, (B) $PGD_2$, (C) $PGF_{2\alpha}$, (D) 6-keto$PGF_{1\alpha}$, (E) $TxB_2$, (F) $LTB_4$ and (G) $LTC_4/D_4/E_4$). The black bars represent the control situation and hatched bars represent the situation after exposure to ozone. Values represent mean ± SEM ($n = 5$). *indicates $p < 0{\cdot}05$. (N.D. means not detectable).

constituents in BAL fluid may be caused by the ozone-induced oxidation of unsaturated fatty acids in lipids and susceptible amino acids in proteins, which results in an alteration of the biological membrane properties. In addition to the increased membrane permeability an influx of inflammatory cells from the blood stream into the alveolar spaces was shown in our histological studies. After exposure to 3 ppm ozone for 2 h only a desquamation of the bronchiolar epithelial layer was observed whereas after exposure to 1 ppm ozone for 12 h an infiltration of neutrophilic granulocytes could be seen. However, the bronchiolar epithelium remained intact.

Cell differentiation performed in guinea-pig broncho-alveolar lavage (BAL) fluid after exposure to these ozone exposure protocols shows comparable results (data not shown). Exposure to 1 ppm ozone for 12 h resulted in an increased amount of neutrophilic granulocytes (represented as percentage of the total amount of cells) compared with the control situation as well as compared with the situation where the animals were exposed to 3 ppm ozone for 2 h. These current results are supported by a number of both human[13,25] as well as animal studies. A variety of animal species has been studied in order to examine the ozone-induced injury at tissue level. Ozone-induced tissue neutrophilia was first demonstrated by Castleman *et al.*[26] in the bronchiolar wall of Rhesus monkeys after a 4 h exposure to 0.8 ppm ozone. Also in mongrel dogs a neutrophilic inflammation was observed in the tracheal and bronchial mucosa[27] already 1 h after exposure to 2.1 ppm ozone for 2 h which was accompanied by an ozone-induced hyperresponsiveness. In a study where guinea pigs were exposed to 3 ppm ozone during 2 h (identical to our acute exposure protocol) the time course of histological changes was examined.[28] In agreement with our findings no

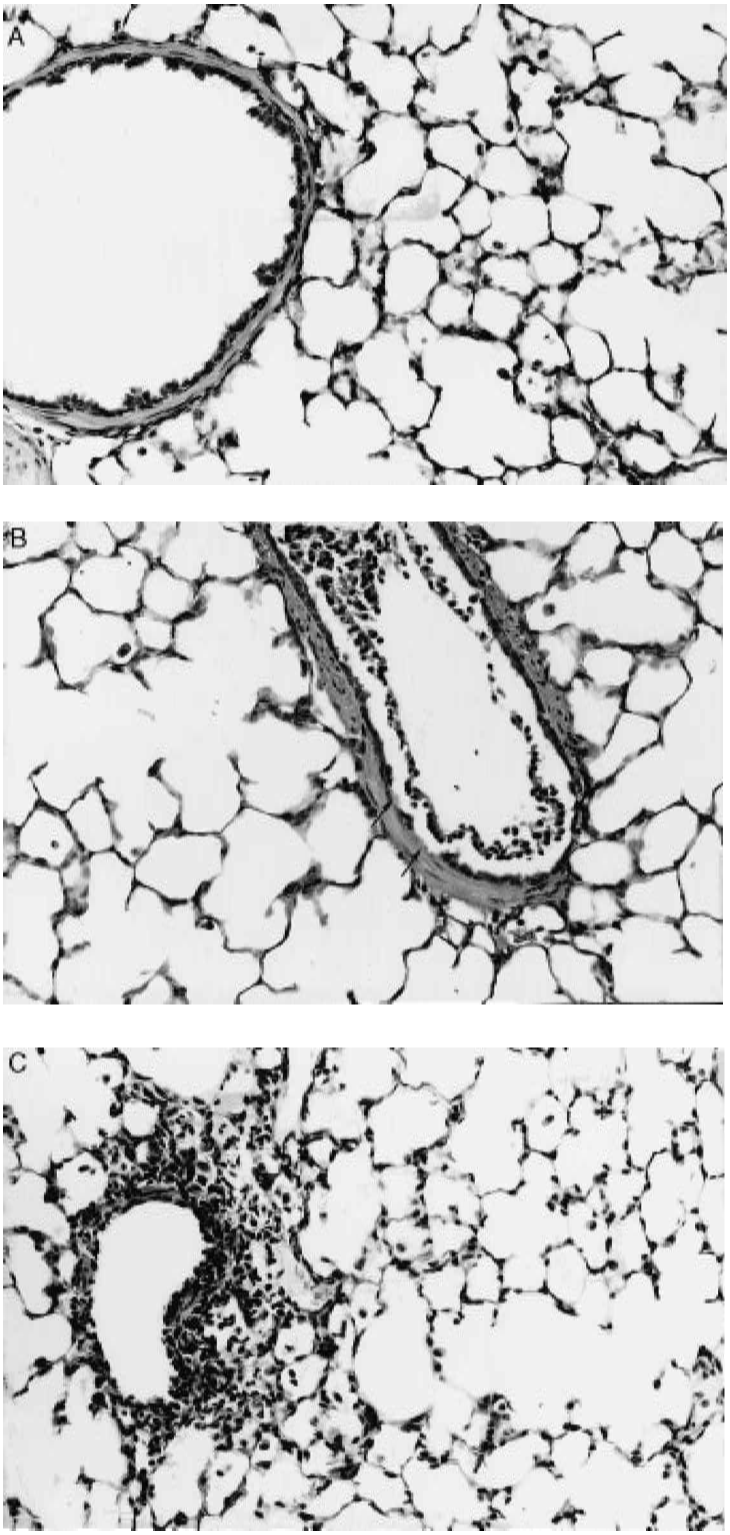

FIG. 2. Histological and morphological effects of ozone exposure on guinea-pig lung tissue. (A) Control guinea-pig lung with a bronchiole and expanded alveoli; (B) guinea-pig lung after exposure to 3 ppm ozone for 2 h: desquamation of bronchiolar epithelial cells resulted in a naked basement membrane. The lumen is filled with these epithelial cells and (C) guinea-pig lung after exposure to 1 ppm ozone for 12 h: a bronchiolitis of polymorphonuclear and mononuclear cells is shown with a centriacinar inflammation (HE, 180×).

inflammatory effect was observed immediately after exposure but at 6 h after exposure an increase in neutrophilic granulocytes was measurable peaking at 2 days post-exposure. Also after an exposure to 2 ppm ozone for 4 h a rapid accumulation of polymorphonuclear leukocytes (PMNs) in guinea-pig lung interstitial and airway spaces was observed.[5] The inflammatory response declined to control values within 24 h in lung interstitium whereas the increased amount of inflammatory cells measured in BAL fluid remained elevated for 3 days. Very recently a study of Sun and Fan Chung[29] also showed an increased number of neutrophils after single as well as after repeated exposure (exposure on 4 successive days) to 3 ppm ozone for 3 h.

The neutrophilic granulocyte is a potential source of a wide variety of mediators, including potent lipid mediators like prostaglandins, thromboxanes, leukotriene $B_4$ and PAF which might contribute to altered airway responses and/or exacerbation of the inflammatory response.[11] In the current study it was shown that after exposure to 3 ppm ozone for 2 h a significant increase in the release of $PGF_{2\alpha}$, $PGE_2$, $PGD_2$ and 6-keto$PGF_{1\alpha}$ (the stable endproduct of $PGI_2$) in guinea-pig BAL fluid was observed. After exposure to a lower concentration over a longer period of time (1 ppm ozone for 12 h) an additional increased release of $LTB_4$ was measured. No effects were observed concerning the release of $TxB_2$ (stable end product of $TxA_2$) and $LTC_4/D_4/E_4$.

Although no neutrophilic inflammatory response was observed after exposure to 3 ppm ozone for 2 h, an increased release of some of the lipid mediators was perceived. This observation suggests that the increase in prostaglandins is not coming from the influx of inflammatory cells, but from cells that are present in the airways under normal conditions, possibly the alveolar macrophages or airway epithelial cells.[30]

It has been shown that $LTB_4$ is a predominant neutrophil chemoattractant[31,32] which is present in alveolar macrophages and alveolar epithelial cells[9,30] and is thought to be responsible for initiating the inflammatory response after ozone exposure.[33] The 5-lipoxygenase pathway in neutrophils selectively generates $LTB_4$ upon stimulation with a variety of stimuli.[30,34] This could explain the observation that after exposure to 1 ppm ozone for 12 h, when an inflammatory response is present, a three-fold increase in the release of $LTB_4$ was observed whereas after exposure to 3 ppm ozone for 2 h no effects were measurable.

In our study it was shown that ozone exposure did not affect the release of $LTC_4/D_4/E_4$. Comparable results were found in a series of experiments where humans were exposed to 0.10 ppm ozone or 0.08 ppm ozone for 6.6 h with moderate exercise (40 l/min) and where BAL was performed 18 h after exposure.[25] No effect was observed in the release of $LTB_4$ although a marked inflammatory response was present. The authors suggest that the time course between ozone exposure and BAL measurements (18 h) may be responsible for this observation, since neutrophils are already attracted to the lung as early as 3 h following ozone exposure.[13] It is possible that $LTB_4$ is present in BAL fluid shortly after ozone exposure. Although no clear indication can be found in the literature it may be expected that comparable with the prostaglandin release[17–20] in the case of leukotriene release a species difference may also account for these observed differences between guinea pig and human studies.

$LTB_4$ itself is not able to contract or relax the airways but it seems able to induce airway hyperresponsiveness by the release of $TxB_2$.[31] After $LTB_4$ inhalation an influx of neutrophils into the airways was observed but also a striking increase in $TxB_2$ in lavage fluid in dogs. This increased airway responsiveness was prevented by pretreatment with the thromboxane synthase inhibitor OKY-046 whereas the inhibitor did not change the amount of inflammatory cells after $LTB_4$-inhalation.[31]

Surprisingly, our study did not show any increase in the release of $TxB_2$ although an increased release of $LTB_4$ was present after exposure to 1 ppm ozone for 12 h. The amounts of $TxB_2$ measured in our experiments are, compared with the other eicosanoids, rather high. This might suggest that the neutrophils are not the major source of $TxB_2$ in our experimental set-up and that basal release of $TxB_2$ from other cell types in the airways[35] exceeds the release from neutrophils. On the other hand, the release of $TxB_2$ from thrombocytes may also account for the observed effects since these cells produce very large amounts of $TxB_2$[36,37] and this might overwhelm the ozone-induced changes in the $TxB_2$ release.

In summary, we have shown in this study that the inflammatory response after exposure to ozone strongly depends on ozone concentration and exposure time. LDH seems to be the most sensitive marker for ozone-induced tissue damage whereas the exudation of albumin and protein is only measurable after exposure over a longer period of time. This exudation is accompanied by a neutrophilic inflammatory response and a subsequent increase in the $LTB_4$ release in guinea-pig BAL fluid. The other mediators remained unchanged ($TxB_2$ and $LTC_4/D_4/E_4$) or were already increased after exposure to 3 ppm ozone for 2 h in the absence of an inflammatory response ($PGE_2$, $PGD_2$, $PGF_{2\alpha}$ and 6-keto-$PGF_{1\alpha}$). The precise role of these eicosanoids in the ozone-induced changes in airway reactivity in our experimental set-up and exposure protocol requires further investigation.

### References

1. Mustafa MG. Biochemical basis of ozone toxicity. *Free Rad Biol Med* 1990; **9**: 245–265.
2. O'Byrne PM. Leukotrienes, airway hyperresponsiveness and asthma. *Ann NY Acad Sci* 1988; 282–288.
3. Devlin RB, McDonnell WF, Becker S, *et al.* Time-dependent changes of inflammatory mediators in the lungs of humans exposed to 0.4 ppm ozone for 2 h: a comparison of mediators found in bronchoalveolar lavage fluid 1 and 18 h after exposure. *Toxicol Appl Pharmacol* 1996; **138**: 176–185.
4. Devlin RB, Folinsbee LJ, Biscardi F, *et al.* Inflammation and cell damage induced by repeated exposure of humans to ozone. *Inhal Toxicol* 1997; **9**: 211–235.
5. Schultheis AH, Basset DJP. Inflammatory cell influx into ozone-exposed guinea pig lung interstitial and airway spaces. *Agents Action* 1991; **34**: 270–273.
6. Schultheis AH, Bassett DJP. Guinea pig lung inflammatory cell changes following acute ozone exposure. *Lung* 1994; **172**: 169–181.
7. VanBree L, Marra M, Rombout PJA. Differences in pulmonary biochemical and inflammatory responses of rats and guinea pigs resulting from daytime or nighttime, single and repeated exposure to ozone. *Toxicol Appl Pharmacol* 1992; **116**: 209–216.
8. Devillier P, Bessard G. Thromboxane $A_2$ and related prostaglandins in airways. *Fundam Clin Pharmacol* 1997; **11**: 2–18.
9. Barnes PJ, Chung KF, Page CP. Inflammatory mediators and asthma. *Pharmacol Rev* 1988; **40**: 49–84.
10. Austen KF, Soberman RJ. Perspectives on additional areas for research in leukotrienes. *Ann NY Acad Sci* 1988; 11–25.
11. Kay AB, Corrigan CJ. Eosinophils and neutrophils. *Br Med Bull* 1992; **48**: 51–64.
12. Weissmann G. Prostaglandins as modulators rather than mediators of inflammation. *J Lipid Med* 1993; **6**: 275–286.
13. Seltzer J, Bigby BG, Stulbarg M, *et al.* $O_3$-induced change in bronchial reactivity to methacholine and airway inflammation in humans. *J Appl Physiol* 1986; **60**: 1321–1326.
14. Janssen LJ, O'Byrne PM, Daniel EE. Mechanism underlying ozone-induced in vitro hyperresponsiveness in canine bronchi. *Am J Physiol* 1991; **261**: L55–L62.
15. Coffey MJ, Wheeler CS, Gross KB, Eschenbacher WL, Sporn PHS, Peters-Golden M. Increased 5-lipoxygenase metabolism in the lungs of human subjects exposed to ozone. *Toxicology* 1996; **114**: 187–197.
16. Holroyde MC, Norris AA. The effect of ozone on reactivity of upper and lower airways in guinea pigs. *Br J Pharmacol* 1988; **94**: 938–946.
17. Olgletree ML, Allen GT. Interspecies differences in thromboxane receptors: studies with thromboxane receptor antagonists in rat and guinea pig smooth muscles. *J Pharmacol Exp Ther* 1992; **260**: 789–794.
18. Norman P, Cuthbert NJ, McKenniff MG, Gardiner PJ. The thromboxane receptors of rat and guinea pig lung. *Eur J Pharmacol* 1992; **229**: 171–178.
19. Aharinejad S, Franz P, Firbas W. Prostaglandin synthesis of the trachea in rats and guinea pigs. *Acta Histochem* 1990; **89**: 81–84.
20. Holtzman MJ, Hansbrough JR, Rosen GD, Turk J. Uptake, release and novel species-dependent oxygenation of arachidonic acid in human and animal airway epithelial cells. *Biochem Biophys Acta* 1988; **963**: 401–413.
21. Marra M, Rombout PJA. Design and performance of an inhalation chamber for exposing laboratory animals to oxidant air pollutants. *Inhal Toxicol* 1990; **2**: 187–204.
22. Doumans BT, Biggs HG. Determination of serum albumin. In: Cooper GR (ed.) *Standard Methods of Clinical Chemistry*. New York: Academic Press, 1972; 175–188.
23. Dumler K, Hanley QS, Baker C, Luchtel DL, Altman LC, Koenig JQ. The effects of ozone exposure on lactate dehydrogenase release from human and primate respiratory epithelial cells. *Toxicol Lett* 1994; **70**: 203–209.
24. Aris RM, Christian D, Hearne PQ, Kerr K, Finkbeiner WE, Balmes JR. Ozone-induced airway inflammation in human subjects as determined by airway lavage and biopsy. *Am Rev Respir Dis* 1993; **148**: 1363–1372.
25. Devlin RB, McDonnell WF, Mann R *et al.* Exposure of humans to ambient levels of ozone for 6.6 hours causes cellular and biochemical changes in the lung. *Am J Respir Cell Mol Biol* 1991; **4**: 72–81.
26. Castleman WL, Dungworth DL, Schartz LW, Tyler WS. Acute respiratory bronchiolitis: an ultrastructural and autoradiographic study of epithelial cell injury and renewal in Rhesus monkeys exposed to ozone. *Am J Pathol* 1980; **98**: 811–840.
27. Holtzman MJ, Fabbri LM, O'Byrne PM, *et al.* Importance of airway inflammation for hyperresponsiveness induced by ozone. *Am Rev Respir Dis* 1983; **127**: 686–690.
28. Murlas CG, Roum JH. Sequence of pathologic changes in the airway mucosa of guinea pig during ozone-induced bronchial hyperreactivity. *Am Rev Respir Dis* 1985; **131**: 314–320.
29. Sun J, Fan Chung K. Airway inflammation despite loss of bronchial hyperresponsiveness after multiple ozone exposures. *Respir Med* 1997; **91**: 47–55.
30. Holtzman MJ. Arachidonic acid metabolism; implications of biological chemistry for lung formation and disease. *Am Rev Respir Dis* 1991; **143**: 188–203.
31. O'Byrne PM, Leikauf GD, Aizawa H, *et al.* Leukotriene $B_4$ induces airway hyperresponsiveness in dogs. *J Appl Physiol* 1985; **59**: 1941–1946.
32. Sibille Y, Reynolds HY. Macrophages and polymorphonuclear neutrophils in lung defense and injury. *Am Rev Respir Dis* 1990; **141**: 471–501.
33. Lewis RA, Austen KF. The biologically active leukotrienes: biosynthesis, metabolism, receptors, functions and pharmacology. *J Clin Invest* 1984; **73**: 889–897.
34. Borgeat P, Samuelsson B, Arachidonic acid metabolism in polymorphonuclear leukocytes: effects of ionophore A23187. *Proc Natl Acad Sci USA* 1979; **76**: 2148–2152.
35. Morley J, Bray MA, Jones RW, Nugteren DH, Van Dorp DA. Prostaglandin and thromboxane production by human and guinea pig macrophages and leukocytes. *Prostaglandins* 1979; **17**: 730–736.
36. Gresele P, Deckmyn H, Nenci GG, Vermylen J. Thromboxane synthase inhibitors, thromboxane receptor antagonists and dual blockers in thrombotic disorders. *Trends Pharmacol Sci* 1991; **12**: 158–163.
37. Hamberg M, Svensson J, Samuelsson B. Thromboxanes. A new group of biologically active compounds derived from prostaglandin endoperoxides. *Proc Natl Acad Sci USA* 1975; **72**: 2994–2998.

**Received 15 August 1997;**
**accepted in revised form 22 August 1997**

## References

[PDF_00656] Aharinejad S., Franz P., Firbas W. (1990). Prostaglandin synthesis of the trachea in rats and guinea pigs.. Acta Histochem.

[PDF_00672] Aris R. M., Christian D., Hearne P. Q., Kerr K., Finkbeiner W. E., Balmes J. R. (1993). Ozone-induced airway inflammation in human subjects as determined by airway lavage and biopsy.. Am Rev Respir Dis.

[PDF_00630] Barnes P. J., Chung K. F., Page C. P. (1988). Inflammatory mediators and asthma.. Pharmacol Rev.

[PDF_00704] Borgeat P., Samuelsson B. (1979). Arachidonic acid metabolism in polymorphonuclear leukocytes: effects of ionophore A23187.. Proc Natl Acad Sci U S A.

[PDF_00679] Castleman W. L., Dungworth D. L., Schwartz L. W., Tyler W. S. (1980). Acute respiratory bronchiolitis: an ultrastructural and autoradiographic study of epithelial cell injury and renewal in rhesus monkeys exposed to ozone.. Am J Pathol.

[PDF_00644] Coffey M. J., Wheeler C. S., Gross K. B., Eschenbacher W. L., Sporn P. H., Peters-Golden M. (1996). Increased 5-lipoxygenase metabolism in the lungs of human subjects exposed to ozone.. Toxicology.

[PDF_00611] Devlin R. B., McDonnell W. F., Becker S., Madden M. C., McGee M. P., Perez R., Hatch G., House D. E., Koren H. S. (1996). Time-dependent changes of inflammatory mediators in the lungs of humans exposed to 0.4 ppm ozone for 2 hr: a comparison of mediators found in bronchoalveolar lavage fluid 1 and 18 hr after exposure.. Toxicol Appl Pharmacol.

[PDF_00676] Devlin R. B., McDonnell W. F., Mann R., Becker S., House D. E., Schreinemachers D., Koren H. S. (1991). Exposure of humans to ambient levels of ozone for 6.6 hours causes cellular and biochemical changes in the lung.. Am J Respir Cell Mol Biol.

[PDF_00668] Dumler K., Hanley Q. S., Baker C., Luchtel D. L., Altman L. C., Koenig J. Q. (1994). The effects of ozone exposure on lactate dehydrogenase release from human and primate respiratory epithelial cells.. Toxicol Lett.

[PDF_00710] Gresele P., Deckmyn H., Nenci G. G., Vermylen J. (1991). Thromboxane synthase inhibitors, thromboxane receptor antagonists and dual blockers in thrombotic disorders.. Trends Pharmacol Sci.

[PDF_00713] Hamberg M., Svensson J., Samuelsson B. (1975). Thromboxanes: a new group of biologically active compounds derived from prostaglandin endoperoxides.. Proc Natl Acad Sci U S A.

[PDF_00692] Holtzman M. J. (1991). Arachidonic acid metabolism. Implications of biological chemistry for lung function and disease.. Am Rev Respir Dis.

[PDF_00683] Holtzman M. J., Fabbri L. M., O'Byrne P. M., Gold B. D., Aizawa H., Walters E. H., Alpert S. E., Nadel J. A. (1983). Importance of airway inflammation for hyperresponsiveness induced by ozone.. Am Rev Respir Dis.

[PDF_00658] Holtzman M. J., Hansbrough J. R., Rosen G. D., Turk J. (1988). Uptake, release and novel species-dependent oxygenation of arachidonic acid in human and animal airway epithelial cells.. Biochim Biophys Acta.

[PDF_00634] Kay A. B., Corrigan C. J. (1992). Asthma. Eosinophils and neutrophils.. Br Med Bull.

[PDF_00701] Lewis R. A., Austen K. F. (1984). The biologically active leukotrienes. Biosynthesis, metabolism, receptors, functions, and pharmacology.. J Clin Invest.

[PDF_00686] Murlas C. G., Roum J. H. (1985). Sequence of pathologic changes in the airway mucosa of guinea pigs during ozone-induced bronchial hyperreactivity.. Am Rev Respir Dis.

[PDF_00607] Mustafa M. G. (1990). Biochemical basis of ozone toxicity.. Free Radic Biol Med.

[PDF_00653] Norman P., Cuthbert N. J., McKenniff M. G., Gardiner P. J. (1992). The thromboxane receptors of rat and guinea-pig lung.. Eur J Pharmacol.

[PDF_00695] O'Byrne P. M., Leikauf G. D., Aizawa H., Bethel R. A., Ueki I. F., Holtzman M. J., Nadel J. A. (1985). Leukotriene B4 induces airway hyperresponsiveness in dogs.. J Appl Physiol (1985).

[PDF_00609] O'Byrne P. M. (1988). Leukotrienes, airway hyperresponsiveness, and asthma.. Ann N Y Acad Sci.

[PDF_00649] Ogletree M. L., Allen G. T. (1992). Interspecies differences in thromboxane receptors: studies with thromboxane receptor antagonists in rat and guinea pig smooth muscles.. J Pharmacol Exp Ther.

[PDF_00622] Schultheis A. H., Bassett D. J. (1994). Guinea pig lung inflammatory cell changes following acute ozone exposure.. Lung.

[PDF_00619] Schultheis A. H., Bassett D. J. (1991). Inflammatory cell influx into ozone-exposed guinea pig lung interstitial and airways spaces.. Agents Actions.

[PDF_00638] Seltzer J., Bigby B. G., Stulbarg M., Holtzman M. J., Nadel J. A., Ueki I. F., Leikauf G. D., Goetzl E. J., Boushey H. A. (1986). O3-induced change in bronchial reactivity to methacholine and airway inflammation in humans.. J Appl Physiol (1985).

[PDF_00698] Sibille Y., Reynolds H. Y. (1990). Macrophages and polymorphonuclear neutrophils in lung defense and injury.. Am Rev Respir Dis.

[PDF_00689] Sun J., Chung K. F. (1997). Airway inflammation despite loss of bronchial hyper-responsiveness after multiple ozone exposures.. Respir Med.

[PDF_00636] Weissmann G. (1993). Prostaglandins as modulators rather than mediators of inflammation.. J Lipid Mediat.

